# Research on Health and Thermal Comfort of Unit-Type Student Apartments in the Western China Science and Technology Innovation Harbor

**DOI:** 10.3389/fpubh.2022.850107

**Published:** 2022-02-25

**Authors:** Zanshe Wang, Yue Wu, Zhaoying Jia, Qi Gao, Zhaolin Gu

**Affiliations:** School of Human Settlement and Civil Engineering, Xi'an Jiaotong University, Xi'an, China

**Keywords:** unit-type apartment, individual independent space, thermal comfort, field measurement, the western China science and technology innovation harbor, questionnaire survey

## Abstract

**Background:**

In China, the collective living mode of “multiple people living in one room” is widely used in college students' dormitories; the space environment has high personnel density and poor thermal comfort and healthy environment. The unit-type apartments were completed in the western China science and technology innovation harbor that considers personal independence and public activity spaces.

**Aim:**

The purpose of this study is to explore the applicability, thermal comfort, healthy environment, and the correlation of influencing variables of the new unit-type apartment. Especially the influence of physical parameters on personal space under heating in winter and air conditioning in summer.

**Method:**

The field investigations and questionnaires to conduct a personal study of architectural space, healthy environment, and thermal comfort were carried out, and the measurement tests of the building's physical environment were carried out in winter and summer semesters.

**Results:**

The questionnaires survey shows that the privacy of the unit apartment is satisfactory, and the independent learning and communication is increased. The field measurement results show that due to the narrow space of the private room, the floor radiant heating mode still forms a hot and dry thermal environment in winter, and the temperature fluctuates significantly after the air conditioner is turned on in summer.

**Conclusions:**

The unit-type apartments align with young students' physiological and psychological characteristics and the behavioral aspects of postgraduates, with high comprehensive satisfaction up to 80%. However, the indoor thermal environment quickly fluctuates due to the narrow independent space. Moreover, 90% of the window ventilation ratio shows that it has become the main measure to actively regulate the indoor climate, which is beneficial to students' health but increases energy waste and further aggravates the fluctuation of the thermal environment. More refined regulations should be executed for heating in winter and air conditioning in summer.

## Introduction

In the background of the SARS virus pandemic in 2003 and the COVID-19 pandemic in 2019, research on people gathering areas and indoor healthy and comfortable environments in buildings has received more attention. Indoor environment parameters play an important role in human physiology, psychology, and health (1). The college's dormitory is a space complex for students to rest, study, communicate and store private belongings. It is also a residential and intensive activity area for groups of people, its space utilization and comforts directly affect health and quality of life.

In the past 20 years, undergraduates and postgraduates in China have shown rapid growth every year (2). Meanwhile, student accommodation has gradually evolved from an old building with tight space to a direction of standardization, comfort, individualization, and diversification. The facilities and spatial layout of the student dormitory are also gradually improving; the function of the dormitory is slowly changing from the original single bedtime to complex operations such as sleeping, studying, and washing (3). Table 1 shows the most common student dormitory in China, with an area of 15–20 m^2^; the accommodation types are 8-person rooms and 4-person rooms. It has the characteristics of high population density and is relatively closed (4). The healthy environment, air quality, and thermal comfort of the dormitory are generally poor, and the dormitory still belongs to the centralized living mode of “multiple people living in one room” in a corridor-style collective apartment. Although functional zoning and some structural adjustments have been made, it still lacks attention and protection to students' personalities, habits, privacy, health, and psychology.

**Table 1:** Typical dormitory and its characteristics.

**Item**	**8-person room**	**4-person room**	**2-person room**
Dormitory picture	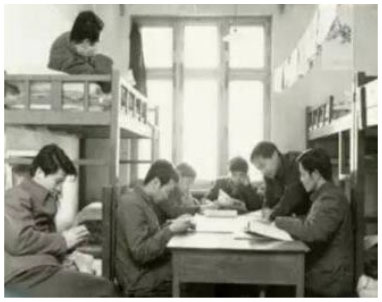	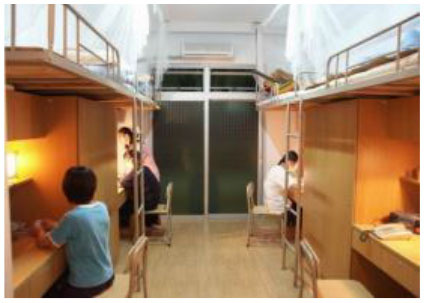	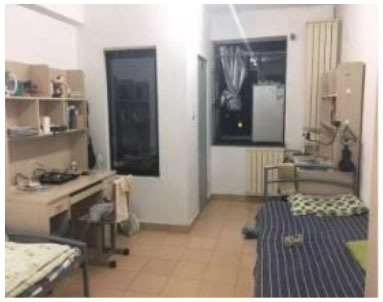
Form	Bunk beds	Upper bed, lower table	Standard living room
Feature	Space congestion, spatial overlap, poor privacy	Space division, privacy, and independent space	Ph.D. student, privacy, and separate space
Function	Rest, socialize	Rest, socialize, study	Rest, scientific research
Comfort	Poor	Moderate	Comfortable

The studies on the health and thermal comfort environment of student dormitories in the literature are mainly carried out from climatic factors, energy utilization, ventilation, sleep and health, psychology, etc. Most methods used are questionnaire surveys, field measurements, and thermal comfort analysis discussions based on ASHRAE standards. For example, research on human thermal comfort before and after heating supply in dormitories in the cold region (5–7), research on thermal comfort of student dormitories in summer (8, 11, 25, 26), research on thermal comfort throughout the year (12, 13), research on natural ventilation in dormitories (14, 16, 17), research on sleeping environment and sleep quality of dormitory students (18), the influence of student emotions on thermal comfort (19), research on air conditioning equipment in hot and humid areas (20), power consumption research under thermal comfort (27), PMV and energy consumption issues (21, 28), etc. The detailed research contents and methods of literatures are shown in Table 2.

**Table 2:** Literature review on thermal comfort of university dormitories or apartments.

**Research topic**	**Research method**	**Valid questionnaires**	**Country or region**	**Highlighted results**
Human thermal comfort during heating and non-heating periods (5)	Field test and questionnaire survey	426 questionnaires from three buildings	Shenyang, China	The dormitory temperature is high in heating period. Reducing the heating temperature for energy-saving.
Adaptive thermal comfort in the severe cold area (6)	Field test and questionnaire survey	632 questionnaires	Harbin, China	As a long-lasting thermal exposure to the heating environment, the thermal adaptability to cold climate undermined, and sensitive to indoor temperature fluctuations.
Nationality dierences in thermal comfort during winter (7)	Field test and subjective questionnaire	Two universities dormitories Japanese:128 International:172	Toyohashi, Japan	Japanese students were more sensitive of and more critical about their indoor environment as opposed to the internationals.
Nationality dierences in thermal comfort during summer (8)	Field test and subjective questionnaire	Two universities dormitories Japanese:183 International:237	Toyohashi, Japan	The occupants' acceptance for thermal stress was invariably above 90%. Japanese students were more sensitive of and more critical about their indoor environment.
Summer thermal comfort and adaptation of dormitories (9)	Field test and questionnaire survey	Two universities 437 questionnaires	Changsha, China	Changing clothes, opening windows and using fans are the three main adaptive behaviors
Thermal comfort of dormitory in summer (10)	Field survey of enclosure structure	None	Wuhan, China	Increase ventilation to improve heat dissipation efficiency, sunshade components and low radiation glass
Thermal comfort of dormitory in summer (11)	Field test and questionnaire survey	113 questionnaires	Tianjin, China	Passive cooling transformation of buildings, increasing indoor ventilation
Thermal comfort of dormitory (12)	Field test and questionnaire survey	3–4 times per month, 9 months Cumulative 1572 questionnaires	Chongqing, China	In summer season, people prefer somewhat cooler condition than neutral, but in the winter season people prefer somewhat warmer condition than neutral.
Thermal comfort requirements for campus dormitories (13)	Questionnaire survey	Cumulative 1,219 questionnaires	Taiwan, China	The students shared similar neutral temperature, preferred temperature and upper limit of acceptable temperature in both ventilated and air-conditioned dormitories.
Thermal comfort in naturally ventilated dormitory (14)	Questionnaire survey and environmental testing	467 questionnaires	Changsha, China	The adaptive behaviors of clothing adjustment and air velocity adjustment were closely correlated to indoor temperature.
Adaptive comfort in air conditioned dormitories (15)	Field test and questionnaire survey	479 questionnaires	Changsha, China	Long-time living in hot-humid regions enhanced occupants' adaptation.
Thermal comfort and adaptive behaviors in naturally ventilated residential buildings (16)	Questionnaire survey and field testing	225 questionnaires	Singapore	Increasing the indoor air velocity and opening the door/windows for cross ventilation; reducing clothing insulation; higher indoor air velocities were associated with greater satisfaction.
Thermal comfort in naturally ventilated classrooms during the summer season (17)	Questionnaire survey and field testing	Cumulative 900 questionnaires	Jaipur, Rajasthan, India	Principal adaptive opportunities available to students were clothing level change, opening windows and regulating ceiling fans
Thermal comfort in sleeping environment (18)	Questionnaire survey and field testing	10 students (6 male; 4 female)	HongKong, China	Females selected a bedding system with higher total thermal resistance than males for a sense of comfort and better sleep quality
Effect of emotion state on people's thermal comfort (19)	Questionnaire survey and field testing	18 students	Qingdao, China	Emotions have significant influence on subjects' physiological parameters, which affects subjects' perception to thermal comfort.
Thermal sensation and comfort in air-conditioned dormitory (20)	Questionnaire survey and field testing	467 questionnaires	Changsha, China	Head exerted the highest influence on overall thermal sensation, followed by calf and foot, then the influences of chest and back were comparatively lower.
Thermal comfort interventions based on occupants' needs (21)	Questionnaire survey	72 questionnaires	Crete, Greece	According to the survey results, the energy-saving transformation scheme of old students' buildings is put forward.
Thermal adaptation in university classrooms and dormitories (22)	Questionnaire survey	30 juniors	Harbin, China	Clothing insulation in classrooms was bigger than in dormitories, and students felt more comfortable in dormitories than in classrooms.
Students' comfort and adaptation in dormitories in humid subtropical climatic area in winter (23)	Questionnaire survey and field testing	30 volunteer subjects	Chongqing, China	Staying for longer periods in regions with a colder climate in winter, improved students' adaptability to lower temperature, closely correlated to behavioral and psychological processes
Thermal comfort analysis of a dormitory with evaporative cooling air conditioner (24)	Field test and questionnaire survey	Cumulative 200 questionnaires	Xi'an, China	Improve the thermal comfort of human body by reducing the amount of clothes, increasing the air flow speed in the dormitory.

From the above literatures, there are many studies on the thermal comfort of university dormitories in China, these university dormitories are located in severe cold areas, cold areas, hot summer and cold winter areas, hot summer and warm winter areas, etc. Compared with foreign student apartments or dormitories, the common feature of Chinese student dormitories is a single unit of 8-person or 4-person, with an area of about 20 square meters. Moreover, due to the similarity of dormitory units and student characteristics, the number of questionnaires is hundreds of levels, with certain representativeness and robustness.

Although the above literature rarely involves unit-type apartments, its research methods and conclusions can inspire.

At present, the concentrated living mode of Chinese college student dormitories is relatively small and densely populated; its structural form determines that it cannot meet the requirements of individualized space and living comfort.

The unit apartment is a new type of student apartment with diversified functions, humanized living environment, and a scientific management model; it has the advantages of both unit houses and apartment houses, its space is moderate, the layout is standard and compact, and it is economical and practical, the public use area is increased, and the indoor living facilities are complete, with solid development potential (29–37).

Although the unit apartment can provide students with diversified life and spiritual needs in terms of function and space, it is still in the conceptual design stage in China in the past years; there are few similar research reports on the unit apartment, especially for postgraduate students, the thermal comfort of apartments, and the thermal environment at home and abroad.

In September 2019, the Western China Science and Technology Innovation Harbor (hereinafter referred to as the “Innovation Harbor”) was completed. More than 20,000 postgraduate students enrolled in 2019, 2020, and 2021. This study takes the unit-type student apartment on the Innovation Harbor campus as an example, using questionnaires, on-site testing methods, and combined with SPSS software, to measure in the winter semester and summer semester, to explore the applicability, spatial integration, and thermal comfort of this new apartment type, and analyze the relationship between the environmental physical parameters and indoor health and thermal comfort in the apartment.

Therefore, Compared with the literature shown in Table 2, starting from the students' spatial needs, psychological characteristics, and physical environmental needs, this study focuses on the applicability and thermal comfort of the new unit-type apartment buildings rarely used in colleges and universities of China, to provide reference significance for the improvement and development of college apartment in the future.

## Overview of the Unit-Type Apartments

### Layout of Student Apartment

The Innovation Harbor is located in Xixian District, Xi'an, China, with 34.3° north latitude, 108.93° east longitude, and an altitude of 397 m. There are three dormitory groups with a total area of 333,000 square meters. The Innovation Harbor has a warm temperate semi-humid continental monsoon climate with four distinct seasons. Winters are cold, windy, rainy and snowy; springs are warm, dry, and windy; summers are hot and rainy, and autumns are cool. The average annual temperature is 13.0–13.7°C, the average temperature in January is −1.2–0.0°C, the hottest July month with an average temperature of 26.3–26. 6°C, the annual precipitation is 522.4–719.5 mm. Annual sunshine hours 1,646.1–2,114.9 h, the annual dominant wind to the northeast wind.

**Figure 1:**
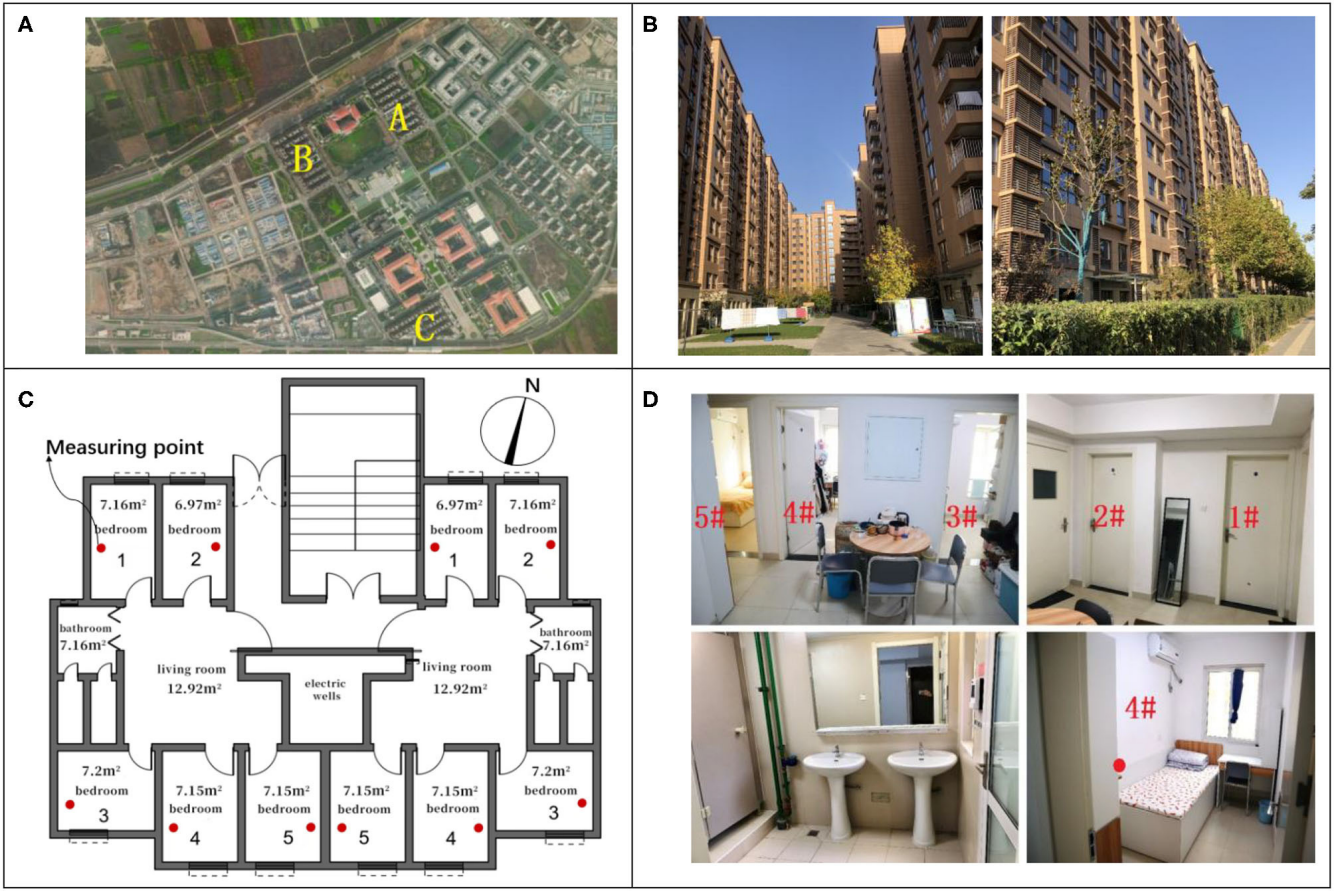
Unit-type student apartment in the innovation harbor. **(A)** Three apartments. **(B)** Apartment Photos. **(C)** Apartment plan. **(D)** Internal photos.

Figure 1 shows the location map, the external map, the floor plan, and the internal physics of the unit apartment. The overall orientation is north-south and can accommodate more than 20,000 students. All the building adopts unit apartments with two-unit, three-unit, and multiple-unit models. The 11-floor small high-rise design has two households with one elevator. Each unit includes five independent small rooms, a shared living room, and a bathroom, as shown in Figure 1D. The separate room is about 7 square meters, with a single bed, a set of desks and chairs, a wardrobe, and windows that can be opened independently, as shown in 4# of Figure 1D. The shared living room is about 13 square meters, with a sizeable public table and five chairs. The bathroom is equipped with two toilets, two sinks, and a shower room. For the convenience of statistics and the test marking, the number of each room in the apartment is shown in Figure 1C.

### Envelope Structure and Energy Supply Mode

The apartment is a shear wall structure, the main wall is an aerated concrete infill wall, and the outside of the wall is wrapped with a layer of 10 cm thick thermal insulation board. The outer window adopts a single frame double glass steel window. The roof surface adopts extruded foam insulation board, SBS waterproof coiled material, and a cement mortar protective layer.

Each room in the apartment is equipped with a split-type air conditioner used for cooling and temperature adjustment in summer; students can adjust the temperature by themselves. In winter, the radiant floor heating mode is adopted. The heating source is from the non-interfering geothermal heating system of medium and deep geothermal energy, “take the heat but not water,” and the heating temperature can be controlled by adjusting the valve opening in each room.

## Questionnaire Survey

### Questionnaire Survey Content

In ASHRAE standard, the definition of human thermal comfort is the conscious state of people's satisfaction with the thermal environment (38, 39). It is determined by human physiology and psychology. Two main factors are affecting human thermal comfort: (1) environmental factors: air temperature, air humidity, average radiation temperature, and air velocity; (2) Human factors: Clothing and activity (40, 41). Some environmental factors will cause local discomfort to the human body, such as the feeling of blowing, the large temperature difference between the head and feet, the asymmetry of radiation temperature (42, 43), etc. Therefore, The applicability, spatial integration, and subjective feelings of the unit apartment were investigated through questionnaires.

1. Subjective questionnaire survey. Including the basic information about the apartment and the respondent, the respondents' evaluation of the apartment, communication among members, and usage of the shared living room; the adaptability of apartment space and environment, space utilization, personal life, and study habits, etc.
2. Thermal environment investigation. Take temperature, humidity, air quality, thermal comfort, etc., as the survey indicators (44, 45), as shown in Table 3. The subjective expression of the thermal sensation perception [−3, −2, −1, 0, 1, 2, 3] is expressed as “cold”, “cool”, “slightly cool”, “neutral”, “slightly warm”, “warm”, and “hot” (46); the humidity sensation perception [−1, 0, 1] is expressed as “damp”, “comfortable”, and “dry”; the thermal comfort perception [4–0] is expressed as “unbearable”, “great uncomfortable”, “uncomfortable”, “slightly uncomfortable”, and “comfortable”; and the air quality perception [−2, −1, 0] is expressed as “very bad”, “poor”, and “good”. The central threshold range of the thermal sensation and the humidity sensation is based on the “Indoor air quality standard” of GB/T 18883-2002 (47). Since the indoor air quality involves not only the physical environment parameters, but also the particulate, chemical and bacterial contaminants, it is difficult to describe it with only one index, therefore, combined with the characteristics of the unit apartment, the fresh air volume is used as the subjective evaluation standard (47), that is, when there is no fresh air, the air quality is very bad, when the fresh air volume is less than 30 m^3^/h/person, the air quality is poor, and when the fresh air volume is great than 30 m^3^/h/person, the air quality is good.

**Table 3:** Thermal environment questionnaire survey.

**Index**	**Option indicator**	**Central threshold range**	**Index**	**Option indicator**	**Central threshold**
Thermal sensation scal	−3, −2, −1, 0, 1, 2, 3	Winter: 16–24°C Summer:22–28°C	Thermal comfort scal	4, 3, 2, 1, 0	Subjective feeling
Humidity sensation scal	−1, 0, 1	Winter: 30–60% Summer:40–80%	Air quality scal	−2, −1, 0	Fresh air 30 m^3^/h/person

### Survey Results

Since almost all students first settled in the unit-type apartment from an 8-room or a 4-Room undergraduate dormitory, their psychological feelings, space utilization, and thermal environment feelings have a great sense of contrast and effectiveness, the questions involved in the survey are diverse, so the designed questionnaire is not a standard Richter scale questionnaire. Therefore, the reliability and validity of the questionnaire were not tested. However, the content design of the questionnaire covers as much as possible the influencing factors of indoor thermal comfort of unit-type apartments, such as the objective factors (floor, room orientation, and living life, etc.), subjective physiological feelings such as indoor temperature, humidity, and wind feeling, and other potentially influencing factors such as lighting, noise, and air quality.

In total, 368 valid samples were collected, including 200 in winter and 168 in summer. The scope of the survey involves three apartment communities of the whole school and more than 100 apartments in 20 unit buildings. Among the subjects surveyed, 54% were male, and 46% were female. Male and female subjects are evenly distributed in the apartments, and 90% are on floors 2–10, and 10% are on the first and top floors. The proportion of north-facing and south-facing rooms of the apartment is uniform and representative. All the situation of the subjects meets the requirements of this survey. The winter and summer thermal comfort surveys were conducted in December 2019 and July 2020.

#### The Questionnaire Survey Results

According to the questionnaire, firstly, the unit apartment form fits the physical and psychological characteristics of young students and the work and life patterns of postgraduate students. More release of students' personality, protection of individual habits and privacy, convenient work and rest, and more autonomy in life and studying. Secondly, the female in the apartment prefer quietness, privacy and are accustomed to closing the door. The male is active and communicates actively in the shared living room.

Moreover, in terms of space utilization, personal belongings are placed in individual rooms. The shared books, exercise equipment, scientific research equipment, etc., are placed in the shared living room. The apartment is an exemplary realization of personal and public spaces classification. The unit apartment provides good architectural applicability and spatial integration in the diversity of spatial elements, the harmony and unity of structural elements, and meeting the individual needs of occupants.

**Figure 2:**
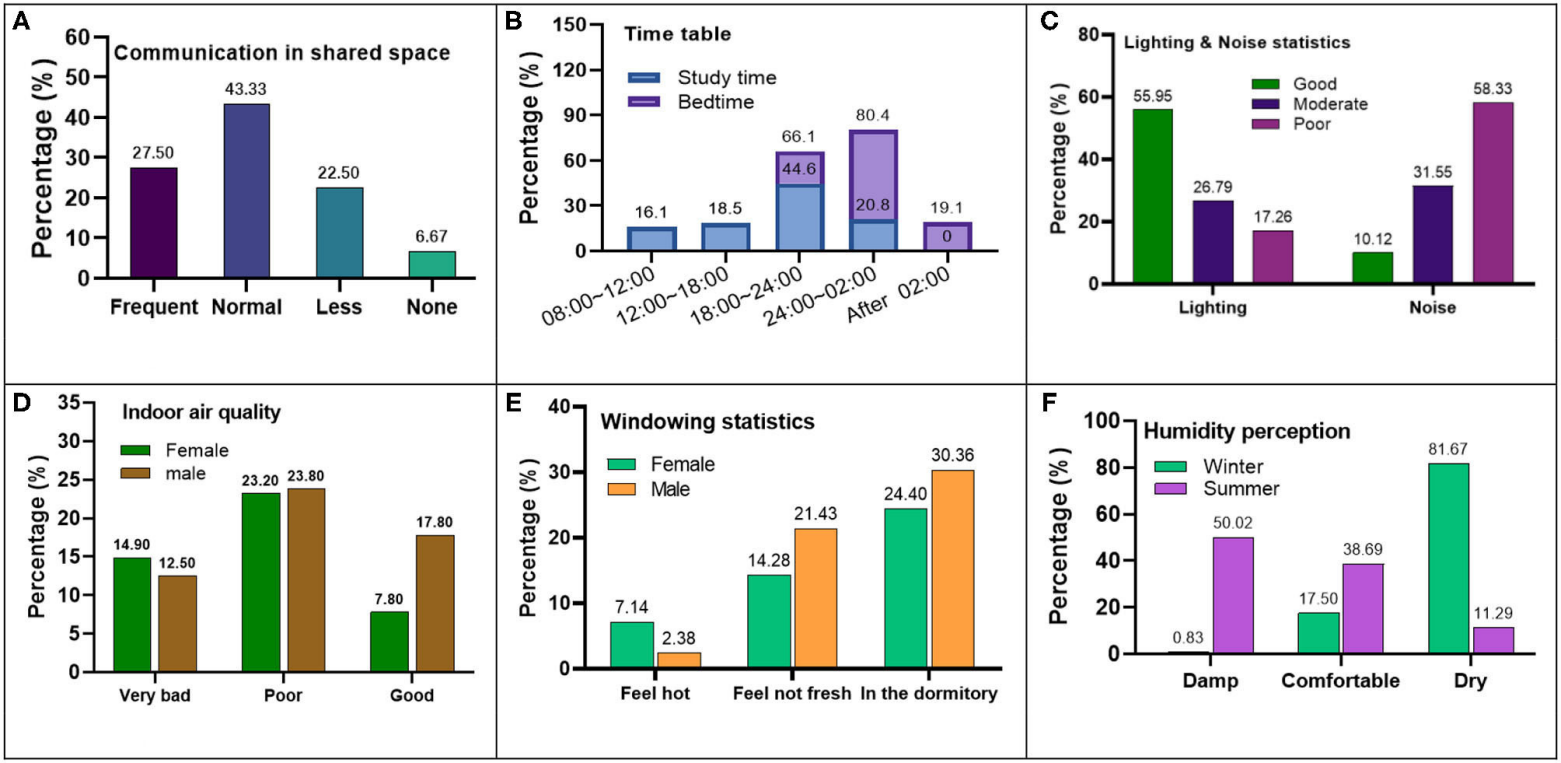
The basic information results of the questionnaire survey. **(A)** Communication. **(B)** Time table. **(C)** Lighting and Noise. **(D)** Indoor air quality. **(E)** Windowing for ventilation. **(F)** Humidity perception.

Besides, the immediate results of the questionnaire survey were shown centrally in Figure 2. Communication is essential for student's mental health and comfort; frequent communication was defined as more than 5 times a day, or more than 10 min per session, 2–5 times means regular communication, 1–2 times means less communication. The result is shown in Figure 2A. The communication in the shared space is active, and generally in the afternoon, dinner time, and evening.

The timetable is shown in Figure 2B; due to the characteristics of postgraduate students and the work convenience, more than 80% of the students sleep later than midnight, and more than 60% of students studying in the apartment at night, and male sleep and study later. It shows the convenience of learning and the diversity of life habits brought by individual independent space.

The light and sound environment is shown in Figure 2C; due to the independent window, most students are satisfied with the natural lighting effect of the dormitory; however, about 60% of the students reckon the noise in the dormitory is loud, which mainly spread from outdoor.

Figures 2D,E are the indoor air quality and ventilation, respectively. Due to the small space of the individual room, most students feel not good about the air quality, and they are used to opening windows for ventilation. It brings significant challenges to the energy consumption of heating in winter and air conditioning in summer.

Figure 2F shows the humidity sensation statistics in winter and summer; due to the low relative humidity in winter in the northwest of the Innovation Harbor, most people feel dry during the heating mode in winter; in summer, the relative humidity general tendency is damp sensation, and female are more sensitive to humidity factors.

#### The Survey Results of Thermal Comfort

**Figure 3:**
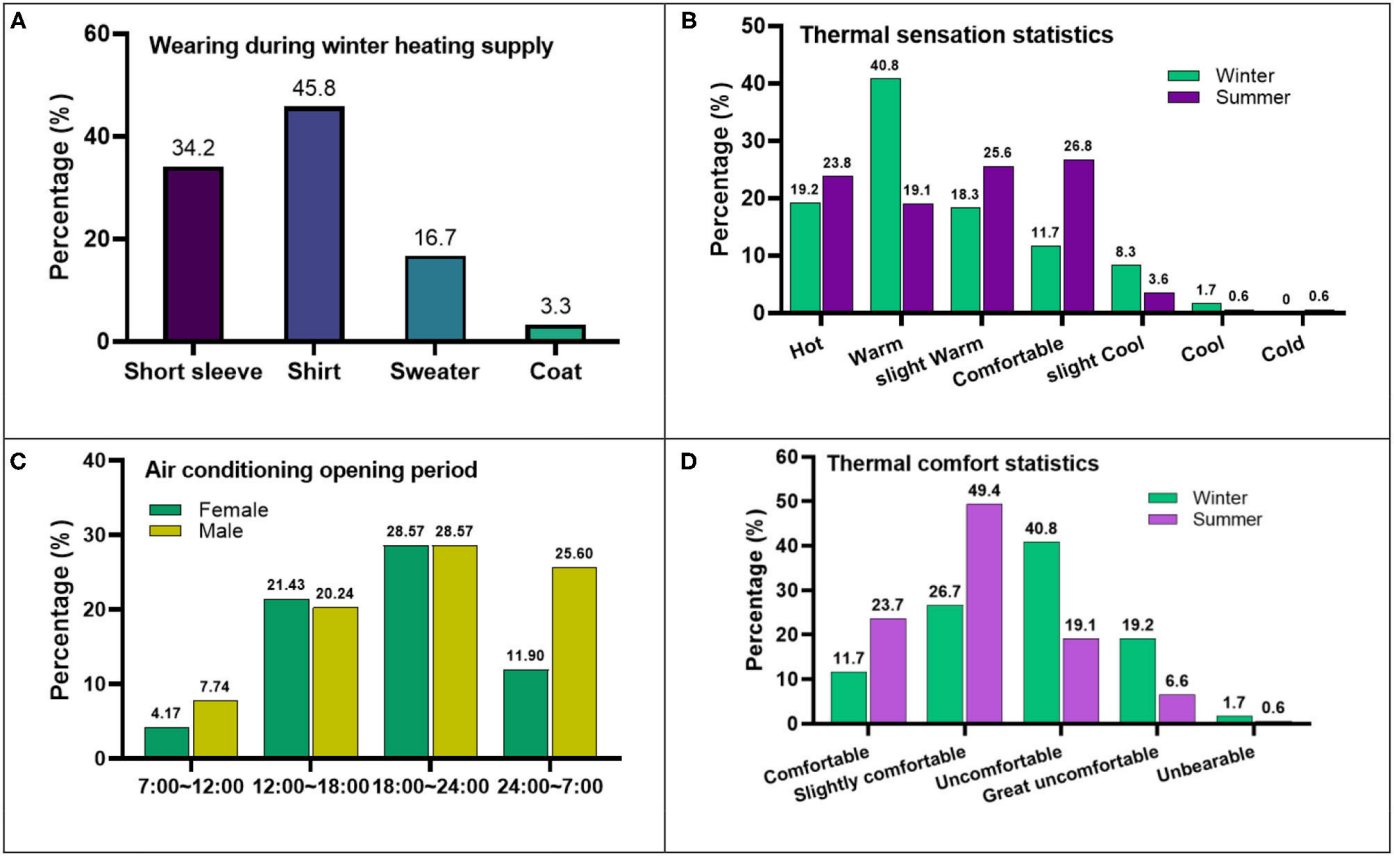
Thermal environment survey results. **(A)** Wearing during winter heating supply. **(B)** Air conditioning opening period. **(C)** Thermal sensation statistics. **(D)** Thermal comfort statistics.

The thermal environment survey results were shown centrally in Figure 3. Firstly, due to the significant temperature difference between indoors and outdoor in winter, personnel clothing wear statistics of indoors in winter is shown in Figure 3A. It inferred that the temperature in the room is generally high during the heating supply period. Secondly, Figure 3B showed the air conditioning opening period in summer, the period of the air conditioner is mainly concentrated at night, and it is consistent with the sleeping and studying period in Figure 2B, and males spend more time on air conditioning than female.

Figure 3C is the thermal sensation statistics in winter and summer. In winter, the indoor thermal sensation is generally high in the floor radiant heating mode, consistent with the clothing worn in Figure 3A. In summer, affected by the summer outdoor environment and window-opening habits, males and females' average TSV (Thermal Sensation Vote) are 1.14 and 1.45, respectively; most of the subjects deviate from the thermal neutral temperature, the indoor thermal sensation are also high.

Figure 3D is the statistical result of thermal comfort. It can be seen that the overall indoor thermal comfort is generally uncomfortable in winter due to the high temperature and low humidity. While in the summer, it is generally a relaxed state, and the gender factor has a weaker influence.

Furthermore, during winter heating, although the indoor temperature can be regulated by the heating valve, few students adopted this method, just opening the window for ventilation, which causes energy waste. This situation puts forward higher requirements for the accurate adjustment of indoor heating.

On the other hand, 90% of students use air conditioning or an opening window for ventilation to adjust the temperature in summer, and 10% choose a fan to cool. Among them, 18:00–24:00 is the main period of air conditioning. More than 85% of students deem that the indoor temperature fluctuates drastically and feels hot and cold alternately with the switch of the air conditioner because of the narrow space of the apartment room; this situation also puts forward higher requirements for the accurate adjustment of indoor cooling.

### The Influence of Different Variables on Thermal Comfort Satisfaction

#### Comparison of Demographic Variables

According to the survey results, the independent sample *T*-test and one-way ANOVA test in SPSS software were used to compare the differences among subjects of gender, floors, orientations, and dormitory groups. The independent sample *T*-test was selected for the gender factor due to its dichotomous. At the same time, the one-way ANOVA test was chosen for the aspects of the building orientation, the floors, and the dormitory groups due to their multi-classified. When the significance values *P* are more significant than 0.05, it indicates no significant difference in this factor. As a result, the significance values *P* of each parameter was calculated and shown in Tables 4, 5. The results of all the significance values *P* are more significant than 0.05, which means that the demographic variables of the gender, the room orientation, the floor, and the dormitory group have no effect on the dormitory satisfaction evaluation and do not need to be used as control variables in subsequent studies on factors affecting subject satisfaction.

**Table 4:** *T*-test for gender differences independent samples.

**Item**	**Male**	**Female**	***T***	***P***
Thermal sensation	1.14 ± 1.346	1.45 ± 1.176	−1.584	0.115
Thermal comfort vote	2.09 ± 0.89	2.10 ± 0.836	−0.119	0.905
Thermal comfort satisfaction	3.42 ± 0.944	3.39 ± 0.975	−0.189	0.851
Humidity feeling	2.13 ± 0.378	2.21 ± 0.414	−1.235	0.219
Expecting temperature	1.28 ± 0.453	1.27 ± 0.447	−0.163	0.871

**Table 5:** One-way ANOVA test of the significance values *P*.

***P***	**Floors**	**Room orientation**	**Dormitory buildings**
Thermal sensation	0.458	0.314	0.142
Thermal comfort vote	0.419	0.277	0.320
Thermal comfort satisfaction	0.323	0.298	0.114

#### Correlation Analysis

The bivariate correlation analysis in SPSS Statistic software was used to obtain the correlations between variables and to measure their prediction or explanatory ability. Due to the different emphasis of questionnaire design in summer and winter, the variables tested are different. The correlation analysis focuses on the variables related to the subjects' thermal comfort satisfaction in summer and the thermal sensation in winter as the bold values shows in Tables 6, 7. As a result, the Pearson correlation coefficients between variables in summer and winter are obtained in Tables 6, 7. When Pearson correlation coefficient *R* > 0, the two variables are positively correlated, and when *R* <0, the two variables are negatively correlated. The value of |R| decides the correlation strength.

**Table 6:** Correlation analysis of variables in summer.

**Pearson correlation coefficient *R***	**Thermal sensation**	**Humidity feeling**	**Thermal comfort vote**	**Thermal comfort satisfaction**	**Air quality**	**Blowing feeling**
Thermal sensation	1					
Humidity feeling	−0.129	1				
Thermal comfort vote	0.464^**^	−0.270^**^	1			
**Thermal comfort satisfaction**	–0.395^**^	0.244^**^	–0.620^**^	1		
Air quality	−0.008	0.270^**^	−0.266^**^	0.236^**^	1	
Blowing feeling	0.201^**^	−0.383^**^	0.290^**^	−0.327^**^	−0.417^**^	1

** *Correlation was significant at 0.01 level (double-tailed)*.

**Table 7:** Correlation analysis of variables in winter.

**Pearson correlation coefficient *R***	**Temperature**	**Expecting temperature**	**Clothing**	**Temperature difference**	**Thermal sensation**	**Humidity feeling**
Temperature	1					
Expecting temperature	−0.122	1				
Clothing	−0.307^**^	0.108	1			
Temperature difference	0.248^**^	−0.249^**^	−0.325^**^	1		
**Thermal sensation**	−0.661^**^	0.241^**^	0.370^**^	−0.325^**^	1	
Humidity feeling	−0.261^**^	0.355^**^	0.164	−0.076	0.273^**^	1

** *Correlation was significant at 0.01 level (double-tailed)*.

In the summer survey, the results shown in Table 6 that the thermal sensation, the humidity feeling, the thermal comfort vote, the air quality, and the blowing feeling are all significantly correlated with the thermal comfort satisfaction. While in the winter survey, the results are shown in Table 7 that the temperature, the expecting temperature, the clothing wear, the temperature difference between morning and night, and the humidity feeling are all significantly correlated with the thermal sensation.

## Physical Parameter Measurements

### Test Instruments and Methods

The test period for the winter semester is from December 25, 2019, to January 7, 2020, and the test period for the summer semester is from July 14, 2020, to August 5, 2020.

Three male apartments and three female apartments are selected as the test objects. The apartments on the 2nd, 4th, and 11th floors were chosen by considering the environmental differences between the low, middle, and top floors. Each apartment contains a south-facing and north-facing room, whose numbers are shown in Figure 1C. The measuring instrument is placed in the room's central area with a height of 1.5 m. The test instruments and their accuracy are shown in Table 8. Outdoor weather parameters are recorded by the automatic weather station (Davis-6162).

**Table 8:** Instruments and accuracy.

**Parameters**	**Instruments**	**Accuracy**
Temperature and Relative humidity	Testo-175H1	±0.4 °C; ±2% RH
Air velocity	Testo-425	±0.03 m/s
CO_2_ concentration	Testo-535	±50 ppm CO_2_

### Test Results

**Figure 4:**
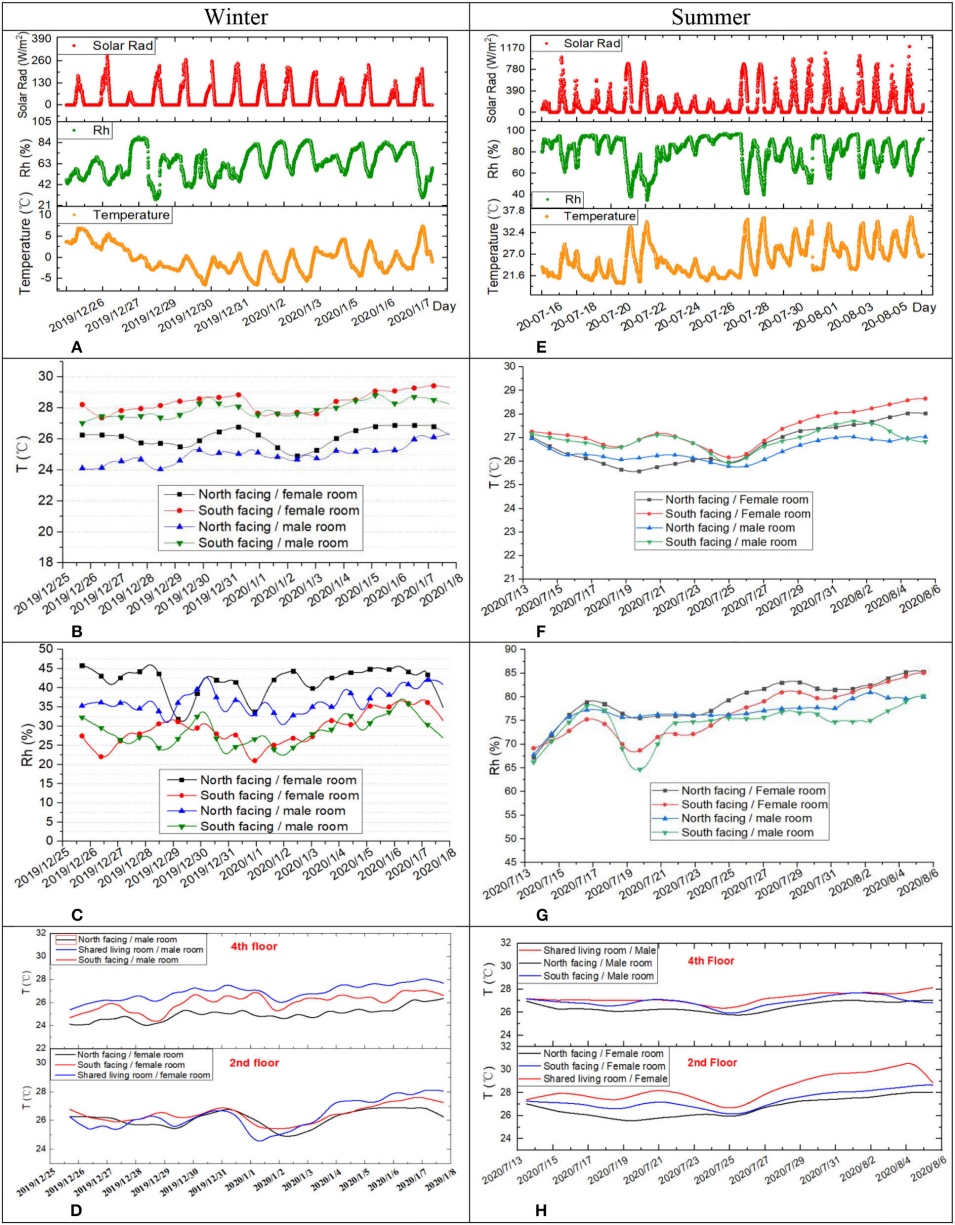
Test results in winter and summer. **(A)** Outdoor parameters during winter test. **(B)** Indoor temperature. **(C)** Indoor relative humidity. **(D)** North, South, and shared living room. **(E)** Outdoor parameters during summer test. **(F)** Indoor temperature. **(G)** Indoor relative humidity. **(H)** North, South, and shared living room.

The test results in winter and summer were shown centrally in Figure 4. As a background meteorological parameter, Figures 4A,E are the outdoor solar radiation, air temperature, and relative humidity parameters during the winter and summer test period, separately.

The winter's 15-day temperature and humidity test data are shown in Figures 4B,C. According to the statistics of the temperature and humidity test data for different floors and room orientations, the temperature distribution is in the range of 24–29°C, which inferred a heat-based room environment. Among the south-facing of male and female rooms, it is similar and relatively high to that of the north-facing room caused by solar radiation, and the temperature of the male room is low than that of the female room due to the windowing habit for ventilation that is shown in Figure 4E. Furthermore, the shared living room temperature is higher than that of other rooms as a whole because of the windowing habit, shown in Figure 4D. On the other hand, the relative humidity distribution in all the rooms is generally lower than 45% shown in Figure 4C; it also accords with the characteristics of room-drying perception survey data Figure 4F.

The 21-day temperature and humidity test data in summer are shown in Figures 4F,G. Similarly, analysis with winter measurements, the temperature distribution is in the range of 25.5–29 °C, which also means a slightly heat-based room environment. The south-facing of rooms is higher than that of the north-facing room, and the temperature of the shared living room is higher than that of other rooms because there is no air conditioning in the shared living room. More importantly, the relative humidity is generally high than 75%, which fits the characteristics of room-damping perception survey data Figure 2F.

In summary, due to the small area of the individual independent room, the physical parameters are easy to mix uniformly and fluctuate greatly; on the whole, the temperature and the relative humidity fluctuation cycle in the apartment is the same as the fluctuation cycle of the outdoor environment, and it is generally preferred to open windows for ventilation in summer.

**Figure 5:**
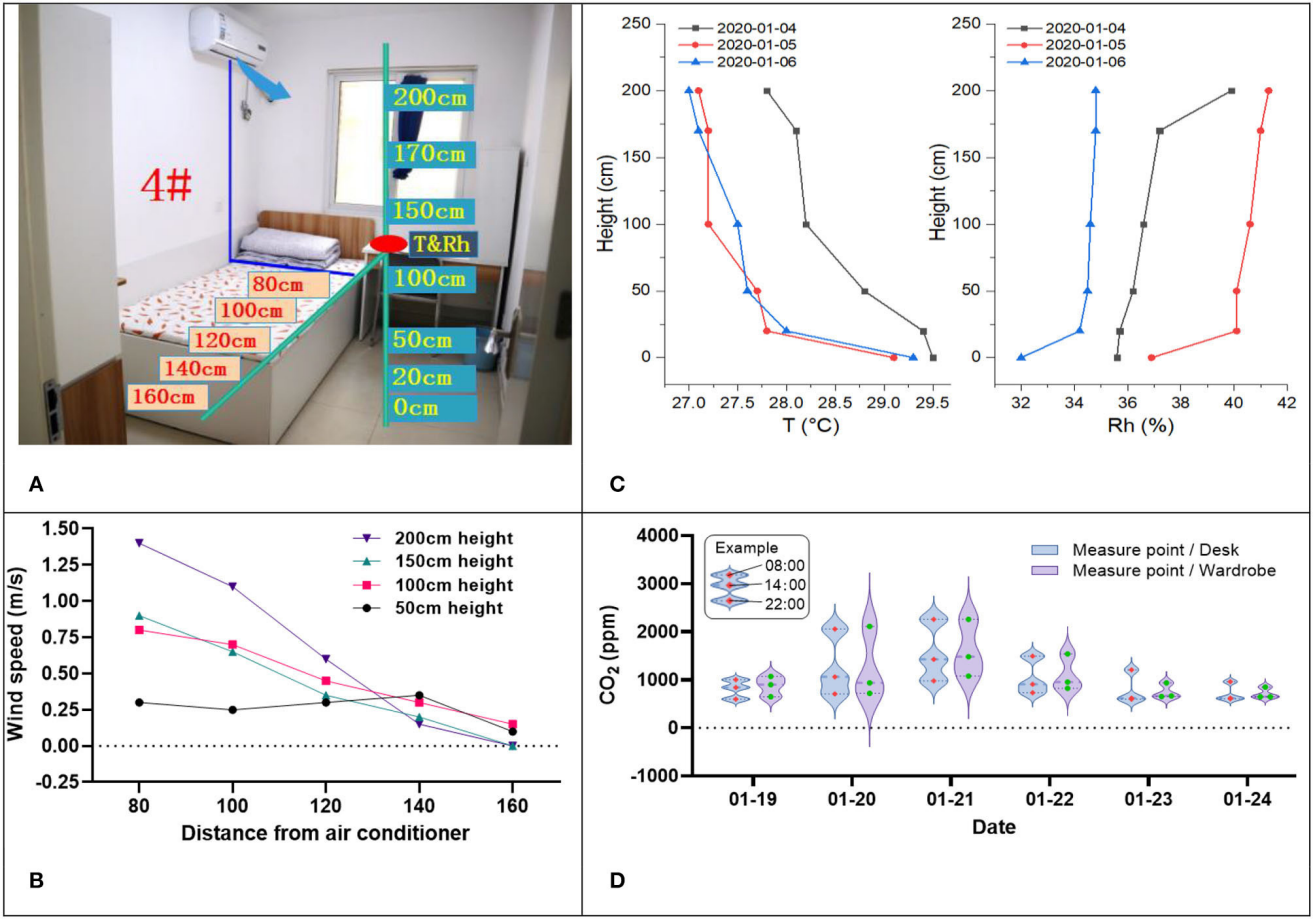
Other test results. **(A)** Indoor sensor arrangement. **(B)** Indoor wind speed in summer. **(C)** T and Rh in the height direction in winter. **(D)** Carbon dioxide concentration.

In addition, the temperature and relative humidity in the vertical height direction, the carbon dioxide concentration, and the indoor wind speed with the air conditioning were measured, as centrally shown in Figure 5.

Figure 5A is the sensor arrangement in the 4# room of a female apartment to test the temperature and the relative humidity in vertical directions, the CO_2_ concentration, and the wind speed with the air conditioning in horizontal and vertical directions.

Figure 5B shows the indoor wind speed at different horizontal distances and vertical heights. In summer, 70% of males and 90% of females chose to set the air outlet of the air conditioner to blow in the direction of 1.8–2m height to avoid the air conditioner blowing directly at the human body. For all that, the wind speed is locally greater than 0.3 m/s due to the small area of the room; the wind speed fluctuates quickly at the range of standing height. The blowing feeling and the varying wind speed in the field of human movement in the room are apparent, producing local discomfort. Therefore, the air supply mode of the air conditioner should be controlled more accurately.

Figure 5C shows the 3 days consecutive measurement results of the temperature and relative humidity in the vertical height direction of the 4# room of a female apartment in winter. Due to the radiant floor heating mode, the temperature distribution in the vertical height direction shows a gradient decreasing trend, which is consistent with the human thermal comfort; the humidity does not change much in the vertical direction. It shows that the indoor temperature of 4# room is much higher than the normal winter comfortable temperature range in winter, which is the same as that of the south-facing room shown in Figure 4B. The reasons are related to the small space of 7 m^2^, and although there is a heat source control valve in each room, few students adjust it.

Figure 5D shows a 6-day measurement result of the CO_2_ concentration in the desk and the wardrobe of 4# room in winter, the CO_2_ concentration is the highest in the morning (8:00), which is caused by the window-closing during sleep at night. While at noon (14:00) and night (22:00), the CO_2_ concentration decreases due to the window opening habit, which also illustrates the importance of open windows for ventilation in small spaces.

## Discussion

### Environment and Space Analysis

The unit apartment of the Innovation Harbor is a new type of student dormitory; compared with the traditional centralized residence model of student dormitory, there are similarities and significant differences. First of all, most of the young students in recent years are growing up in the era of the one-child period; they have grown up in the internet age, emphasizing self-worth, diversified living habits, and an intimate space environment. Moreover, the residents of the unit apartment are mainly postgraduate students, the majors of the postgraduate group are divided, the research direction is meticulous, the study and work styles are more focused, and the schedules are flexible and diverse. Meanwhile, postgraduate students have a solid willingness to gather and communicate.

Therefore, the unit apartment fits young students' physical and psychological characteristics and the features of the study and work at the postgraduate level. The division of individual independent space and public space considers both the individual and gathering characteristics of the living environment.

### Suggestions on the Thermal Environment Control

Nearly 4,000 postgraduate apartments and more than 20,000 postgraduate students live in the Innovation Harbor. Whether heating supply in winter or air-conditioning in summer, there is a great potential for energy consumption and energy-saving. From the perspective of building a healthy and comfortable environment and building energy efficiency, the unit apartment also needs to refine the thermal environment's energy supply and regulation mode.

1. A more comprehensive thermal comfort survey and immense data accumulation are needed, including behavioral characteristics and regularities such as postgraduate study, life, work style, rest habits, subjective thermal sensations, and expected environmental parameters. Students need to be guided to form energy-saving habits, such as opening windows for ventilation, turning down or turning off energy supply equipment when going out, and actively adjusting heating flow or air-conditioning temperature when in the dormitory.
2. According to the variables with a strong correlation with heat feeling and thermal comfort satisfaction, the current situation of influencing factors can be improved in a targeted manner, such as air quality, noise, and wind feeling are variables with strong correlation, which will affect the body's hot and comfortable feel to a large extent.
3. In addition to the shared living room, each room in the apartment is compact and small with an independent window, the indoor thermal and humidity environment is easy to fluctuate.

In summer, firstly, five small rooms in an apartment are equipped with five split air conditioners is inappropriate, it not only increases the initial investment, but also easily causes the fluctuation of environmental parameters, and the frequent start and stop of air conditioning in the whole building is also easy to cause fluctuation to the power network. Since the total area of the apartment is about 50 m^2^, a small central air conditioning system is one of the options for this apartment form, which can be arranged in each small room with air supply outlets and control switches, respectively. On the whole, it can reduce the initial investment and achieve the purpose of energy-saving (48, 49). Moreover, for small spaces, the arrangement and analysis of the airflow field should be carried out to achieve a uniform layout without causing a feeling of blowing at the height of the pedestrian layer, Therefore, the arrangement of the air outlet position requires the simulation calculation and the detailed measurement to achieve indoor comfort (50).

In winter, the radiant floor heating mode is a recognized energy-saving and comfort heating mode that can form a flow field mode with a temperature gradient from the floor to the roof (51). However, as measured in Section Test Results, the simple rated flow mode not only causes the room temperature to be too high but also causes a waste of energy. For winter heating in small spaces, even if the floor heating pipe network was laid, the temperature can be adjusted and controlled through the automatic regulation mode of flow.

Whether heating in winter or air conditioning in summer, both of which require refined energy supply and control modes. The indoor thermal environment can be dynamically measured and adjusted by adding online data collection and intelligent personnel recognition.

## Conclusion

According to the applicability and thermal comfort of the unit apartment in the Innovation Harbor, field investigation, questionnaire survey, and field test in winter semester and summer semester are carried out; the results showed that:

1. The division of personal space and public space in the unit apartment considers both the individual and gathering characteristics of the living environment, which aligns with the physiological and psychological aspects of young students and the behavioral characteristics of graduate students. The thermal comfort is greatly improved, and the overall satisfaction is generally high.
2. Due to the compact size of the rooms with independent windows, the indoor thermal and humid environment parameters are easy to fluctuate both in winter and summer. To improve the thermal comfort and energy-saving characteristics of apartments, more precise environmental adjustment modes are required.
3. Opening windows for ventilation has become the norm because of the small space, and indoor air quality has also become the first adjusting factor. It is necessary to start with big data on student behavior characteristics and regularities, change the traditional energy supply and regulation mode, and create a healthy, comfortable, and energy-efficient apartment environment.

## Data Availability Statement

The original contributions presented in the study are included in the article/supplementary materials, further inquiries can be directed to the corresponding author.

## Author Contributions

ZW: drafting and writing papers. YW: questionnaire survey and experimental measurement. ZJ: questionnaire investigation. QG: experimental measurement. ZG: make important modifications to the paper. All authors contributed to the article and approved the submitted version.

## Funding

This work was financially supported by the National Natural Science Foundation of China (No. 51478386), by the Innovation Chain of Key Industries of Shaanxi Province (No. 2018ZDCXL-GY-10-03), and by the Natural Science Foundation of Zhejiang (No. LQY19E060001).

## Conflict of Interest

The authors declare that the research was conducted in the absence of any commercial or financial relationships that could be construed as a potential conflict of interest.

## Publisher's Note

All claims expressed in this article are solely those of the authors and do not necessarily represent those of their affiliated organizations, or those of the publisher, the editors and the reviewers. Any product that may be evaluated in this article, or claim that may be made by its manufacturer, is not guaranteed or endorsed by the publisher.
